# A new paradigm for retroperitoneal leiomyosarcoma: integrating transcriptomic subtyping and surgical risk stratification for AI-guided drug repurposing

**DOI:** 10.3389/or.2026.1744721

**Published:** 2026-02-04

**Authors:** Nan Jia, Zicheng Bao, Zhidong Zhang, Kaixing Wang, Yong Li

**Affiliations:** The Third Department of Surgery, The Fourth Hospital of Hebei Medical University, Shijiazhuang, China

**Keywords:** artificial intelligence, drug repurposing, precision medicine, retroperitoneal leiomyosarcoma, surgical oncology, transcriptomics

## Abstract

Retroperitoneal leiomyosarcoma (RLMS) remains a major therapeutic challenge because of frequent postoperative recurrence and the limited benefit of current adjuvant therapies. The marked molecular heterogeneity of RLMS and its incompletely characterized oncogenic drivers have hindered the development of effective targeted therapies. This review proposes an integrative framework that combines transcriptomic subtyping with surgical risk stratification to support artificial intelligence (AI)–guided drug repurposing. The delineation of RLMS subtypes and the identification of potential therapeutic targets through transcriptomic analysis are described, including PDGFRα and VEGFA. The AI-guided screening of approved and investigational drug libraries to identify compounds predicted to reverse subtype-specific molecular programs; preclinical studies highlight candidates such as pazopanib and histone deacetylase (HDAC) inhibitors is discussed. Finally, the outline of a personalized strategy is proposed, in which surgical decision-making integrates anatomic risk with molecular signatures to inform the selection of neoadjuvant or adjuvant therapies. Integrating surgical management, multi-omics, and computational pharmacology helps bridge the gap from bench to bedside and, ultimately, improve outcomes for patients with RLMS. In contrast to prior work that addresses molecular subtyping or surgical management in isolation, this review presents an integrative framework that links surgical risk stratification with transcriptomic profiling to enable AI-guided drug repurposing and provides a roadmap for personalized RLMS therapy.

## 1 Introduction

Retroperitoneal sarcomas (RPS) comprise a heterogeneous group of malignancies that pose substantial therapeutic challenges (1). Among RPS, retroperitoneal leiomyosarcoma (RLMS) is a common histologic subtype with aggressive behavior and frequent late presentation, partly due to its deep anatomic location (2–4). Complete surgical resection remains the cornerstone of curative-intent therapy. However, local and distant recurrence is common, and outcomes remain poor for patients with recurrent or unresectable disease (5).

Management of RLMS is complicated by substantial molecular heterogeneity. Recent transcriptomic and genomic studies have reported recurrent molecular alterations in RLMS. These include overexpression of receptor tyrosine kinases (e.g., PDGFRα) and angiogenic factors (e.g., VEGFA), as well as dysregulation of cell-cycle and epigenetic pathways (6, 7).

This molecular diversity contributes to variable clinical behavior and treatment response, underscoring the need for subtype-specific therapeutic strategies. Transcriptomic profiling is well suited for RLMS subtyping for its capture of the functional tumor state, including active gene-expression programs that may be more readily targetable than static genomic alterations (8). Moreover, transcriptomic data can identify activated pathways and candidate drug–gene interactions in a dynamic, clinically actionable manner, complementing genomic and proteomic profiling (9, 10).

The high relapse rate in RLMS is further compounded by the inconsistent benefit of adjuvant radiotherapy and chemotherapy across patient subgroups (11–13). Emerging evidence suggests that molecular features may predict therapeutic benefit. For example, tumors with angiogenesis-high signatures may derive greater benefit from anti-angiogenic agents, whereas tumors with epigenetic dysregulation may be sensitive to histone deacetylase (HDAC) inhibitors (14, 15). These observations highlight the limitations of a uniform treatment approach and reinforce the need for biology-driven, personalized strategies.

To better stratify surgical risk, several tools have been developed, including the SARCO-M model (incorporating tumor size, vascular involvement, and prior resection history) and the RPS surgical complexity score (quantifying operative difficulty based on anatomic involvement) (16–18). These instruments provide a standardized, quantitative basis for preoperative assessment of surgical complexity and postoperative risk, thereby supporting individualized operative planning and multidisciplinary decision-making.

The emergence of precision medicine—enabled by high-throughput multi-omics and advanced artificial intelligence (AI)—offers a promising path forward (19). Recent reviews suggest that AI methods, including machine learning and deep learning, can integrate heterogeneous data (genomic, transcriptomic, and clinical) to identify candidate targets and predict drug response. Such approaches may be particularly valuable for rare, heterogeneous tumors such as RLMS, for which conventional trial designs are often impractical. By leveraging AI, it may be possible to accelerate identification of repurposable drugs tailored to specific molecular subtypes, thereby bridging bench research and clinical application (20, 21). Delineating RLMS molecular subtypes and systematically repurposing existing drugs with AI could enable a new therapeutic paradigm.

Although prior reviews have addressed transcriptomic sarcoma classification, surgical risk modeling in RPS, and AI-guided drug discovery separately, these domains have not been integrated into a cohesive RLMS-specific strategy. This review bridges that gap by proposing an integrative paradigm in which clinical surgical risk and molecular subtyping jointly inform AI-guided therapeutic matching—a holistic approach not yet systematically articulated in the literature.

This review synthesizes a novel approach for RLMS, outlining how the integration of transcriptome-based subtyping with refined surgical risk stratification can create a robust foundation for AI-guided drug repurposing, thereby paving the way for truly personalized therapy.

### 1.1 The clinical landscape and surgical stratification in RLMS

#### 1.1.1 Diagnosis and current therapeutic limitations

Diagnosis of RLMS relies primarily on cross-sectional imaging (CT and MRI) for preoperative evaluation and surgical planning (22, 23). Definitive diagnosis is confirmed on post-resection pathology, which typically shows atypical spindle cells and increased mitotic activity; immunohistochemistry (e.g., SMA, Desmin, and Caldesmon) is supportive (24). Despite advances in surgery, recurrence exceeds 50% within 5 years, underscoring the limitations of local therapy alone (5). The benefit of adjuvant radiotherapy and chemotherapy remains inconsistent, and responses vary across patient subgroups (25). Collectively, these observations support a shift from uniform, empiric management to biology-driven, personalized strategies that target the relevant molecular drivers.

#### 1.1.2 The imperative for surgical risk stratification

Surgical management of RLMS is fraught with challenges, as tumors are often large and involve critical structures like the inferior vena cava (IVC), necessitating complex, multi-organ resections (26). While compartmental resection aiming for negative margins (R0/R1) is endorsed, its impact on long-term survival is debated (27, 28). The complexity of these procedures is compounded in recurrent disease due to altered anatomy and adhesions, leading to significant intraoperative blood loss and morbidity.

Therefore, a risk-stratified surgical approach is needed to balance oncologic control with functional outcomes (29). This approach involves preoperative risk categorization based on established factors, including tumor size, major vascular involvement, and prior resection history (30). Emerging tools, such as the SARCO-M model (31) and the RPS surgical complexity score, provide a quantitative basis for stratification (32). This clinical framework also enables integration of molecular data, supporting the hypothesis that tumors with high-risk surgical features may harbor distinct, targetable molecular profiles.

### 1.2 Decoding RLMS heterogeneity through multi-omics

Given the limitations of clinical and surgical parameters alone in predicting outcomes, there is a pressing need to dissect the biological underpinnings of RLMS aggressiveness and heterogeneity. This is where high-throughput multi-omics technologies offer invaluable insights.

#### 1.2.1 The role of transcriptomics in subtyping

The “omics” revolution—encompassing genomics, transcriptomics, proteomics, and metabolomics—has expanded the analytical scope of cancer biology and enabled more comprehensive molecular characterization. Transcriptomics, particularly RNA sequencing (RNA-seq) (33–35), has become central to molecular subtyping (36), enabling comprehensive assessment of gene-expression programs that reflect tumor cell state (37).

In RLMS, transcriptomic profiling is beginning to reveal molecular diversity. For instance, studies have identified subsets characterized by shared gene expression signatures involving dozens to hundreds of genes, such as those enriched for receptor tyrosine kinase signaling (e.g., PDGFRα) and angiogenic pathways (e.g., VEGFA) (38). The identification of such stable transcriptional clusters—rather than individual gene overexpression—provides a more robust basis for molecular taxonomy and reduces susceptibility to biological variance or spatial heterogeneity within tumors.

#### 1.2.2 From molecular subtypes to druggable targets

The primary utility of molecular subtyping lies in its ability to nominate “druggable” targets. The overexpression of PDGFRα and VEGFA in a significant proportion of RLMS cases logically suggests the potential efficacy of anti-angiogenic agents and multi-kinase inhibitors. Furthermore, transcriptome analysis can uncover dysregulated pathways related to apoptosis evasion, cell cycle dysregulation, and epigenetic remodeling—hallmarks of cancer that are increasingly documented in sarcoma biology. For example, alterations in p53 and RB pathways are common in leiomyosarcoma, and epigenetic modifiers such as EZH2 and HDACs have been implicated in sarcoma progression (39–41). Thus, the translation of a distinct molecular subtype into an actionable treatment hypothesis constitutes the critical bridge connecting omics discovery to tangible therapeutic intervention.

### 1.3 Artificial intelligence as a catalyst for drug repurposing

#### 1.3.1 AI in modern drug discovery

AI is increasingly used in pharmacology to integrate complex biological data and generate predictions of drug efficacy (42). Machine learning and deep learning methods can interrogate large-scale datasets—chemical structures, genomic features, and drug-response profiles—to infer candidate compound–target interactions (43).

The predictive performance of AI platforms (e.g., DLEPS and DeepDRK) depends on the quality, representativeness, and clinical relevance of the training data. These models are often trained on large-scale transcriptomic perturbation resources, such as LINCS L1000, which catalog gene-expression changes induced by thousands of compounds across diverse cell lines (44). Drug-induced transcriptomic responses are strongly context dependent. Therefore, predictions for RLMS may require training or fine-tuning using perturbation data from sarcoma-relevant models (e.g., leiomyosarcoma cell lines and patient-derived xenografts). Recent studies have incorporated lineage-specific data and reported improved performance for drug-sensitivity prediction in sarcoma-relevant settings (45). Validation commonly includes benchmarking against established drug-response datasets, evaluation in independent test sets, and experimental confirmation in preclinical models (46).

In the context of RLMS, a transcriptomic signature defining a PDGFRα-overexpressing subtype can be input into an AI platform (e.g., a DLEPS). The system then screens for compounds predicted to reverse this signature, effectively ‘normalizing’ the gene expression profile. This approach directly nominates drugs that are mechanistically poised to counteract the specific oncogenic drivers identified in the patient’s tumor (47, 48).

Recent reviews describe expanding applications of AI across the oncology pipeline, spanning early target discovery through clinical trial design and optimization. In rare cancers such as RLMS, AI-based approaches may partially mitigate small-sample limitations by integrating multimodal data and leveraging transfer learning from larger, related datasets. Explainable AI (XAI) methods are being developed to improve interpretability of model outputs, which may facilitate clinical translation. As these methods mature and are prospectively validated, they may support data-driven, subtype-specific therapeutic recommendations for RLMS (20, 21).

#### 1.3.2 Application in RLMS drug repurposing

In RLMS, AI-guided drug repurposing offers an efficient, hypothesis-generating strategy. Pazopanib, a multikinase inhibitor with activity against VEGFR, is used for advanced RLMS; however, objective response rates are modest. A transcriptomic signature representing a specific RLMS subtype—preferably derived from primary tumors or representative models—can be used as an input to an AI platform. The platform can screen libraries of approved drugs to prioritize candidates predicted to counteract the dysregulated pathways. To improve clinical relevance, the model should be trained and/or validated using sarcoma-relevant transcriptomic perturbation data. This approach has nominated several candidate compounds. For example, these models have prioritized multikinase inhibitors (e.g., pazopanib) and epigenetic modulators (e.g., histone deacetylase inhibitors) based on predicted alignment with RLMS-associated vulnerabilities, including metastasis- and angiogenesis-related programs (49, 50). These *in silico* predictions provide a hypothesis-generating rationale for preclinical testing in biologically relevant models, such as patient-derived xenografts (PDXs). Therefore, future studies should validate prioritized compounds in RLMS-relevant models (e.g., PDXs and RLMS cell lines) to support translational relevance.

Similarly, olaratumab, an anti-PDGFRα monoclonal antibody, was withdrawn after phase III trials failed to demonstrate a survival benefit. Together, these observations highlight the limitations of empiric targeting in the absence of molecularly informed patient stratification. AI approaches may mitigate this limitation by identifying transcriptomic subsets in which these pathways are dominantly dysregulated, thereby enriching for patients more likely to benefit (51). However, VEGFA expression alone has not been established as a predictive biomarker for VEGF-pathway inhibitors; this underscores the need for multi-gene signatures and functional validation.

#### 1.3.3 Challenges and considerations for AI implementation in RLMS

Although AI-guided drug repurposing is promising, its application to rare tumors such as RLMS presents distinct methodological challenges. First, the scarcity of high-quality, well-annotated transcriptomic datasets from RLMS limits the development and training of robust disease-specific models. Multi-institutional efforts to establish RLMS biobanks with linked clinical annotation and multi-omics data are needed.

Second, the limited interpretability of some models can obscure the biological rationale for drug prioritization and hinder clinical adoption. Explainable AI (XAI) methods are being developed to improve interpretability by linking model outputs to specific pathway-level alterations (52, 53).

Third, because drug-induced expression changes are context dependent, AI-guided hypotheses should be validated in biologically relevant models. Future work should prioritize generating perturbation datasets in RLMS-derived cell lines and PDXs to refine model training and improve prediction accuracy.

AI implementation in rare tumors such as RLMS is further constrained by limited sample size (“small data”). Unlike common cancers, RLMS lacks large, harmonized datasets required for robust model development and training. Collaborative initiatives, including federated learning and multi-institutional data sharing, may be required to address this bottleneck. Integrating radiomics and pathomics with transcriptomic data may strengthen predictive signals, particularly when sample sizes are limited. Such multimodal approaches can improve model generalizability and clinical utility in RLMS (20).

### 1.4 Data scarcity and alternative strategies for model robustness

Scarcity of high-quality, well-annotated RLMS transcriptomic datasets remains a key bottleneck for developing reliable models. Given the rarity of RLMS, conventional biobanking alone may be insufficient to achieve sample sizes needed for robust machine learning. Several complementary strategies may mitigate this limitation. These include: (1) multi-institutional aggregation of existing datasets with harmonized clinical and molecular annotation; (2) data augmentation (e.g., generative adversarial networks [GANs] and synthetic data generation) with safeguards for biological plausibility; (3) transfer learning, in which models pre-trained on larger related datasets (e.g., other soft-tissue sarcomas) are fine-tuned using available RLMS data; and (4) multimodal integration (e.g., proteomic, metabolomic, and radiomic features) to enrich predictive signals when transcriptomic sample size is limited. Combined with RLMS-focused biobanking, these approaches may improve model generalizability and support translation of AI-guided hypotheses toward clinical evaluation.

Despite these challenges, integrating AI with transcriptomic subtyping provides a pragmatic framework to prioritize personalized therapeutic hypotheses. As data resources expand and methods improve in transparency and validation, AI may become an important component of the RLMS therapeutic development workflow.

### 1.5 An integrated framework for personalized RLMS management

**FIGURE 1:**
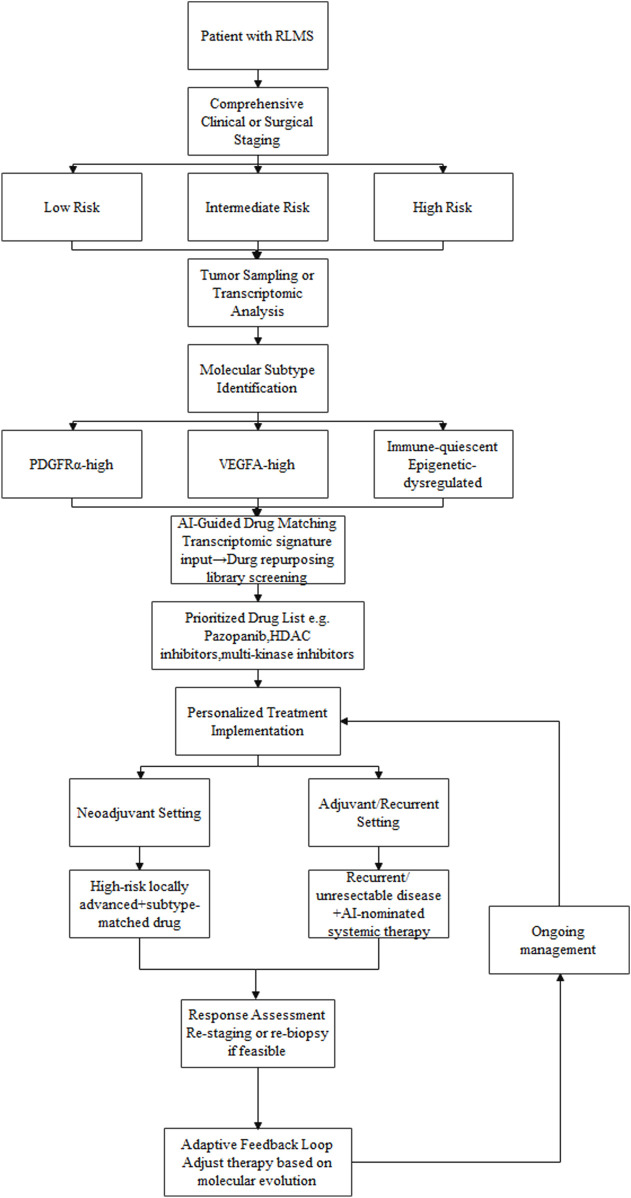
A conceptual framework for integrating clinical risk stratification, transcriptomic subtyping, and AI-guided drug repurposing in RLMS management:The workflow begins with comprehensive clinical and imaging assessment to stratify surgical risk. Tumor samples undergo transcriptomic profiling to define molecular subtypes (e.g., PDGFRα-high, VEGFA-high). These molecular signatures are processed through an AI-based drug prediction platform, which screens repurposing libraries to prioritize candidate drugs (e.g., pazopanib, HDAC inhibitors). The output is integrated with the patient^´^s clinical risk profile to guide personalized treatment decisions, including neoadjuvant therapy for high-risk locally advanced disease or systemic therapy for recurrent/unresectable cases. Response assessment and potential re-biopsy enable an adaptive feedback loop, allowing therapy to be adjusted based on molecular evolution.

We propose a convergent framework in which surgical risk stratification and molecular subtyping jointly inform therapeutic decision-making, supported by AI. As depicted in Figure 1, the framework is designed as an iterative workflow with feedback across clinical, molecular, and treatment layers:

Comprehensive clinical and surgical assessment: Cross-sectional imaging is used to define tumor extent and estimate operative risk, assigning the patient to a prespecified risk stratum (e.g., low, intermediate, high).

Molecular profiling: Tumor tissue undergoes transcriptomic profiling to assign a molecular subtype (e.g., PDGFRα-high, VEGFA-high, immune-quiescent).

AI-guided therapeutic matching: The molecular subtype and associated expression features are entered into an AI-based drug-prediction model to generate a prioritized list of repurposing candidates.

Personalized Treatment Implementation: AI-guided candidate therapies are integrated with the clinical risk profile to inform selection of neoadjuvant or adjuvant strategies.

For example, a patient with high surgical risk and locally advanced disease who exhibits a VEGFA-high subtype could be considered for neoadjuvant therapy with an AI-prioritized anti-angiogenic agent (e.g., pazopanib). This rationale reflects the limited response rates of currently used systemic regimens (e.g., doxorubicin, gemcitabine–docetaxel, and trabectedin), which constrains routine use of neoadjuvant therapy in RLMS. The AI-guided strategy aims to prioritize subtype-matched agents with the potential to improve response. The goal is to facilitate technical downstaging while concurrently targeting biological aggressiveness. After therapy, restaging—and, when feasible, repeat biopsy—could inform subsequent treatment selection, enabling an adaptive, feedback-informed workflow. For patients with recurrent, unresectable disease, AI-prioritized regimens may provide biologically grounded systemic options for evaluation. This strategy is intended to align macroscopic anatomic constraints with molecular tumor biology within a single decision framework.

## 2 Discussion and future perspectives

Integrating transcriptomic subtyping, surgical risk stratification, and AI-guided drug repurposing may enable a substantively different approach to RLMS management. This framework supports a dynamic, individualized precision-oncology model in which treatment selection is informed by both molecular and surgical risk features. By prioritizing existing drugs for repurposing, the strategy may shorten development timelines and reduce costs relative to *de novo* drug development. The proposed framework differs from prior work in three respects: (1) it integrates quantitative surgical risk tools (e.g., SARCO-M): with transcriptomic subtypes to inform therapy selection; (2) it emphasizes training and/or validating AI models using RLMS-relevant perturbation data to improve model relevance; (3) it proposes an adaptive management loop in which treatment is iteratively updated based on longitudinal molecular and clinical assessment. Collectively, these elements extend beyond generic precision-oncology frameworks by explicitly accounting for disease-specific surgical constraints and molecular context in therapeutic decision-making.

However, several methodological and translational challenges remain.

1. Data scarcity and model robustness: Establishing large, well-annotated RLMS biobanks with linked clinical and multi-omics data is important but difficult given disease rarity. To address this limitation, future work should prioritize collaborative frameworks (e.g., federated learning) that enable multi-institutional model training without exchange of raw patient-level data. In addition, *in silico* modeling based on pathway-level perturbations may help prioritize candidate drugs when patient-derived training data are limited. Recent AI-focused reviews suggest that hybrid strategies—combining real-world biobanking with computational augmentation—may be a pragmatic route toward more robust and clinically applicable models in rare cancers (21).
2. Algorithm transparency: Limited interpretability of some models motivates development of explainable AI (XAI) methods to improve transparency, support clinical trust, and provide pathway-level biological context (54).
3. Preclinical and clinical validation: Prospective testing of AI-prioritized drugs in physiologically relevant preclinical models (e.g., PDXs) is an important intermediate step. Future clinical trials should be biomarker driven, enrolling patients by molecular subtype to evaluate the efficacy of repurposed agents in appropriately selected populations.

## 3 Conclusion

In conclusion, this review outlines a novel integrative paradigm that converges surgical oncology, multi-omics, and computational pharmacology—an approach that has not been systematically articulated for RLMS. By integrating anatomic risk with molecular vulnerabilities using AI, an actionable roadmap to accelerate hypothesis-driven development of personalized therapies for this malignancy is provided.

RLMS remains associated with limited effective systemic treatment options and poor outcomes. The convergence of advanced omics technologies, AI methods, and refined clinical stratification provides an opportunity to improve therapeutic development and evaluation in RLMS. By developing an integrated disease model spanning operative findings and molecular profiling, the field can systematically prioritize and test repurposing hypotheses for existing drugs. This integrative, AI-guided framework can accelerate generation and evaluation of personalized treatment hypotheses and, with rigorous validation, improve outcomes for patients with RLMS.
